# Factors associated with the plan to pre-lacteal feeding for the first 6 months among Ethiopian mothers: a multilevel mixed effects analysis of 2019 performance monitoring for action Ethiopia

**DOI:** 10.1186/s40795-023-00784-z

**Published:** 2023-11-03

**Authors:** Natnael Kebede, Eyob Ketema Bogale, Amare Zewdie, Tadele Derbew Kassie, Tadele Fentabil Anagaw, Elyas Melaku Mazengia, Sintayehu Shiferaw Gelaw, Eneyew Talie Fenta, Habitu Birhan Eshetu

**Affiliations:** 1 Department of Health Promotion, School of Public Health, College of Medicine and Health Sciences, Wollo University, Dessie, Ethiopia; 2 Health Promotion and Behavioural Science Department, College of Medicine and Health Science, Bahir Dar University, Bahir Dar, Ethiopia; 3 Department of Public Health, College of Medicine and Health Sciences, Injibara University, Injibara, Ethiopia; 4 Department of Public Health, College of Medicine and Health Science, Wolkite University, Wolkite, Ethiopia; 5 Department of Public Health, College of Medicine and Health Science, Debre Markos University, Debre Markos, Ethiopia; 6 Department of Health Promotion and Health Behaviour, Institute of Public Health, College of Medicine and Health Sciences, University of Gondar, PO.Box.196, Gondar, Ethiopia

**Keywords:** Ethiopia, Mothers, Multilevel mixed effects analysis, Pre-lacteal feeding

## Abstract

**Introduction:**

Despite efforts to promote exclusive breastfeeding for the first six months of life, pre-lacteal feeding remains prevalent in Ethiopia. The study will use data from the 2019 Performance Monitoring for Action Ethiopia (PMA-ET), which is a nationally representative survey that collects information on maternal and child health indicators. Therefore, this study aims to identify individual and community-level factors associated with the plan to pre-lacteal feeding for the first six months among mothers in Ethiopia.

**Methods:**

The datasets from the 2019 Performance Monitoring for Action Ethiopia Survey were analyzed in this study, which included 685 mothers from the survey. Stata version 17.0 was used for data analysis. Multi-level mixed-effect logistic regression was utilized to identify individual and community-level factors that are linked with the plan to pre-lacteal feeding. The strength and direction of the association were presented using an adjusted odds ratio with a 95% confidence interval, and statistical significance was declared at a P value less than 0.05.

**Results:**

The study found that factors significantly associated with the plan to pre-lacteal feeding included mothers without higher education (AOR = 2.5, 95% CI: 1.204–1.204), mothers belonging to poor households (AOR = 11.1, 95% CI: 3.482–35.175), and women in clusters with poor wealth status (AOR = 0.2, 95% CI: 0.043–0.509).

**Conclusion:**

As per the findings of the study, both individual and community-level factors were found to influence the decision to practice pre-lacteal feeding. Educational status and household wealth were significant individual-level factors associated with pre-lacteal feeding, whereas community wealth status was a significant community-level factor. To address this issue, it is recommended to focus on increasing the education level of mothers from lower socioeconomic backgrounds and providing education on the benefits of exclusive breastfeeding and the risks associated with pre-lacteal feeding. These efforts can help in reducing the prevalence of this harmful practice.

## Introduction

Pre-lacteal feeding is associated with age, residence, parity, antenatal care, wealth, occupation, education, child’s sex, delivery place, and poor feeding knowledge [1–5]. Ethiopia’s government implements strategies to improve IYCF through health extension programs and NGO partnerships to address inappropriate breastfeeding practices [6, 7].

Pre-lacteal feeds refer to the food given to newborns before the establishment of breastfeeding or before the production of breast milk, usually within the first day of life [8]. Prelacteal feeding persists in developing nations, despite its negative impact on breastfeeding and newborn health [9]. The prevalence of prelacteal feeding varies globally, with rates ranging from 54.6–93.9% [10–13]. In Africa, a significant percentage of mothers (10.8–75.2%) offer prelacteal feeds to their newborns [14–16]. In Ethiopia, various regions have reported high rates of prelacteal feeding practices, ranging from 17 to 45.4% [17–20]. Prelacteal feeds heighten illness risk and hinder bonding, breast milk production, and suckling if given before colostrum, despite its prevalence in developing nations [21].

However, this practice has been associated with negative health outcomes for both the mother and the child. Despite efforts to promote exclusive breastfeeding for the first six months of life, pre-lacteal feeding remains prevalent in Ethiopia. This study aims to identify individual and community-level factors associated with the plan to pre-lacteal feeding for the first six months among mothers in Ethiopia. The study will use data from the 2019 Performance Monitoring for Action Ethiopia (PMA-ET), which is a nationally representative survey that collects information on maternal and child health indicators. The study is unique and different from previous research findings on pre-lacteal feeding in Ethiopia because it specifically focuses on the individual and community-level factors associated with the plan to pre-lacteal feeding for the first 6 months among mothers in Ethiopia. This study uses a multilevel mixed effects analysis approach to analyze data collected from the 2019 Performance Monitoring for Action Ethiopia (PMA-ET) survey, which provides a comprehensive and up-to-date understanding of the factors that influence pre-lacteal feeding practices in Ethiopia. Additionally, this study provides insights into how these factors vary across different communities and regions in Ethiopia, which can inform targeted interventions to improve infant feeding practices and reduce infant mortality rates in the country. Overall, this study contributes new knowledge and insights to the existing literature on pre-lacteal feeding in Ethiopia and can inform policy and practice to improve maternal and child health outcomes in the country.

The findings from this study will provide valuable insights into the factors that influence mothers’ decisions to engage in pre-lacteal feeding practices. This information can be used to develop targeted interventions aimed at promoting exclusive breastfeeding practices and reducing the prevalence of pre-lacteal feeding in Ethiopia. Furthermore, this study will utilize a multilevel mixed effects analysis approach, which allows for the examination of both individual and community-level factors that contribute to pre-lacteal feeding practices. This approach will provide a more comprehensive understanding of the complex factors that influence maternal behaviors related to infant feeding practices.

## Materials and methods

### Study area and data source

The PMA-ET 2019 Ethiopia study employs a two-stage cluster design to gather data on pre-lacteal feeding practices in Ethiopia. The study categorizes residential areas as urban or rural and sub-regions as strata, covering all 11 geographic regions in Ethiopia. The target population for the study is women aged 15–49 years, with approximately 95% of the population residing in five regions: Addis Ababa, Amhara, Oromia, SNNP, and Tigray. The remaining regions are grouped as a sixth synthetic region called “other” due to population distribution disparities and limited resources.

To estimate pre-lacteal feeding rates with a margin of error below 2% at the national level, the study selects 238 EAs for the fourth round sample. The margin of error is set below 3% for urban and rural estimates and below 5% for each of the five regional levels. The study finds that individual-level factors such as educational status and household wealth, as well as community-level factors such as wealth status, are associated with pre-lacteal feeding practices in Ethiopia.

Given these findings, targeted interventions that address these factors should be developed to reduce the prevalence of pre-lacteal feeding. Programs that aim to improve maternal education and financial stability may be effective, as well as community-level interventions that address poverty and inequality. Further research is needed to identify the most effective strategies for improving infant and young child feeding practices in Ethiopia. The secondary data for this analysis were obtained from PMA-ET of 2019 which was found in the PMA portal (https://www.pmadata.org/_2019).

### Variable measurement

The dependent variable for pre-lacteal feeding was divided into two categories: “Yes/No”. Mothers who intended to use pre-lacteal feeding during the interview were labeled as “Yes”, while those who did not use it during the interview were labeled as “No”.

#### Variables at the individual level

include maternal age, educational status, wealth status, Anti-natal care, and desired delivery place.

#### At the community level, variables

include Region, place of residence, community education, and community wealth status.

### Data processing and analysis

Before recording, labeling, and exploratory analysis using Stata/SE version 17.0, data cleaning was conducted to ensure consistency and completeness. Descriptive statistics were used to present frequency distributions in tables and text. To account for potential disparities in geographical strata selection and non-responses, a sample weight was applied.

After confirming the eligibility of the data for multilevel analysis (i.e., Intra-cluster Correlation Coefficient (ICC) greater than 10% (ICC = 63.4%)), a multilevel analysis was performed. Since the PMA-ET data had a hierarchical structure with individuals (level 1) nested within communities (level 2), a two-level mixed-effects logistic regression model was utilized. This model estimated both the independent (fixed) effects of the explanatory variables and the community-level random effects on the plan for Pre-lacteal feeding. The log of the probability of plan to Pre-lacteal feeding was modeled using a two-level multilevel model.

A preliminary bivariable multilevel logistic regression was conducted, selecting variables with a p-value less than 0.25 before creating three models (models 1–3). Following this, four stages were carried out: Model 0 (an empty or null model without explanatory variables), Model 1 (solely individual-level factors), Model 2 (solely community factors), and Model 3 (both individual and community-level factors). The measures of association (fixed-effects) estimate the connections between the likelihood of women planning Pre-lacteal feeding and various explanatory variables, expressed as Adjusted Odds Ratio (AOR) with their corresponding 95% confidence interval. A variable with a p-value less than 0.05 was considered statistically significant. The measures of variation (random effects) were reported using ICC, Median Odds Ratio (MOR), and proportional change in variance (PCV) to assess the variation between clusters.

The ICC demonstrates how community characteristics affect the variation in plans for Pre-lacteal feeding for mothers. A higher ICC indicates that community characteristics are more important in understanding individual variation in these plans. MOR is the median value of the odds ratio between the highest and lowest risk areas, showing how much residential area determines the probability of Pre-lacteal feeding plans for mothers. PCV measures the total variation attributed to individual and area-level factors in the multilevel model. Multicollinearity was checked among independent variables using a standard error cutoff point of ± 2, and no multicollinearity was found. The log-likelihood test was used to assess the goodness of fit of the adjusted final model compared to previous models (individual and community-level adjustments).

## Result

### Socio-demographic characteristics of respondents

The analysis included a total of 685 mothers. Approximately 46.85% of women were aged between 25 and 34 years. Around 54.53% of mothers attended higher education. In terms of household wealth status, 329 (47.96%) women were classified as poor, and 253 (36.91%) mothers preferred a delivery place rather than a health facility.

The cluster was the unit of analysis for community-level factors in this study, which included 238 mothers. Similar to the higher rural population proportion in Ethiopia, 88.9% of the clusters were located in rural areas. Out of the clusters, 254 (37.17%) were from the Oromia regions, while the remaining clusters belonged to three other regions. The study aimed to create community-level factors by combining values from various individual characteristics. As a result, 56.71% of the clusters had lower levels of wealth status based on aggregate values derived from PMA-ET data on the wealth index and pre-lacteal feeding. Additionally, about 52.99% of the clusters had lower educational attainment than those attending higher education (Table 1).

**Table 1:** Distribution characteristics of participants in 2019 PMA-ET.

Category MEDHS, n = 1,916	Weighted n (%)
**Age**	
15–24 years	231 (33.75)
25–34 years	322(46.85)
35–49 years	133 (19.40)
**Educational status**	
Not attending higher education	311(45.47)
attend higher education	374(54.53)
**wealth status**	
Poor	329(47.96)
Middle	134(19.49)
Rich	224(32.55)
**Desired delivery place**	
Not At the Health facility	253(36.91)
At Health facility	432(63.09 )
**antenatal care**	
No	555(81.01)
Yes	130(18.99)
**Place of residence**	
Urban	76(11.10)
Rural	609(88.9)
**Region**	
Tigray	40(5.95)
Afar	12(1.83)
Amhara	138(20.16)
Oromia	254(37.17)
Somali	37(5.64)
Benishangul-Gumuz	5(0.79)
South nation nationalities of people	161(23.57)
Gambella	2(0.26)
Harari	3(0.43)
Addis Ababa	26(3.82)
Dire Dawa	3(0.38)
**Community education status**	
Lower	363(52.99)
Higher	322(47.01)
**Community wealth status**	
Lower	388(56.71)
Higher	297(43.29)

### Pre-lacteal feeding

The proportion of pre-lacteal feeding among mothers was 34% (30.56, 37.66) in 2019.

### Individual and community-level factors associated with pre-lacteal feeding (fixed-effects)

In Model 1, educational status and household wealth were found to be significantly associated with the plan to pre-lacteal feeding at an individual level. In Model 2, no variables were associated with the plan to pre-lacteal feeding at a community level. However, in Model 3, after adjusting for both individual and community-level factors, educational status, household wealth, and community wealth status were found to have a statistically significant association with the plan to pre-lacteal feeding.

The odds of pre-lacteal feeding were 2.5 times higher among mothers who did not attend higher education compared to those who did attend higher education (AOR = 2.5, 95% CI: 1.204–1.204).

Those mothers who belong to the poor wealth of households were eleven times more likely to plan to pre-lacteal feeding as compared to the rich [AOR = 11.1, 95% CI: (3.482, 35.175)]. However, Women in clusters with higher relative poverty levels had an 80% lower likelihood of planning to pre-lacteal feeding [AOR = 0.2, 95% CI :( 0.043, 0.509)] than women in clusters with middle and rich wealth status levels (Table 2).

**Table 2:** Multi-level mixed effect logistic regression on the plan to pre-lacteal feeding among mothers in Ethiopia, 2019 PMA-ET dataset

Individual and community characteristics	Model 0	Model 1 AOR (95% CI)	Model 2 AOR(95%CI)	Model 3 AOR (95% CI)
**Age**				
15–24 years		1	1	1
25–34 years		0.81 ( 0. 449, 1.449)		0.81(0. 448, 1.477)
35–49 years		0. 58 (0. 258,1.306)		0.60(0. 263, 1.381)
**Educational status**				
Not attending higher education		**2.3(1.17,1 4.432)**		**2.5 (1.204, 5.351)**
attend higher education		1	1	1
**wealth status**				
Poor		**3.7 (1.613, 8.655)**		**11.1(3.482, 35.175)**
Middle		1.3(0. 536, 3.244)		2.4 (0.851, 6.967)
Rich		1	1	1
**Desired delivery place**				
Not At the Health facility		1.10(0.576, 2.128)		1.1 (0.604, 2.361)
At Health facility		1	1	1
**antenatal care**				
No		1.1(0.573, 2.153)		1.1(0.568, 2.178)
Yes		**1**	1	**1**
**Place of residence**				
Urban			1.6(0.684, 3.804)	2.4(0.951, 6.116)
Rural		1	1	1
**Region**				
Tigray			1.8(0.041, 77.00)	1.5 (0.028, 83.526)
Afar			8.9(0.146, 553.716)	5.7(0.069, 468.585)
Amhara			0.9(0.022,34.861)	0.6 (0.011, 28.505)
Oromia			0.8 (0.019 29.779)	0.6 (0.012, 28.293)
Somali			7.9 (0.138 460.365)	7.3(0.095, 556.709)
Benishangul-Gumuz			2.7(0.027 263.015)	1.9(0.015, 257.216)
South nation nationalities of people			2.0 (0.051 80.313)	1.5(0.030, 75.737)
Gambella			6.7(0.029,1532.778)	6.3(0.021, 1957.159)
Harari			0.7(0.001,128.766)	0.9(0.003, 222.703)
Addis Ababa			0.9(0.020, 49.052)	1.0(0.016 63.287)
Dire Dawa			1	1
**Community education status**				
Lower			0.84(0.352, 2.001)	0.7(0.243, 1.992)
Higher				
**Community wealth status**				
Lower			1.4(0.574, 3.309)	**0.2(0.043, 0.509)**
Higher			1	1
**Random effects**				
Variance	**5.7**	5.6	5.3	5.1
The intra-cluster correlation coefficient (ICC) in %	**63.4**	62.9	61.7	60.7
Median odds ratio (MOR)	**9.6**	9.5	8.9	8.5
Explained variance (PCV %)	**Reference**	1.8	7	10.5

### Random effect (a measure of variation)

The multilevel logistic regression results for random effects indicated a significant variation in the plan to pre-lacteal feeding across clusters. The cluster’s correlation coefficient revealed that 63.4% of the variation in the plan to pre-lacteal feeding was associated with factors at the community level. The complete model also confirmed a statistically significant variation in the plan to pre-lacteal feeding across communities or clusters. The overall model accounted for approximately 60.7% of the plan to pre-lacteal feeding in clusters. Additionally, the MOR confirmed that contraception is a factor attributed to the community level. In the empty model, the MOR for the plan to pre-lacteal feeding was 5.7 times, indicating differences (clustering) between communities. However, when all factors were included in the model, the unexplained community variation in the plan to pre-lacteal feeding decreased to a MOR of 5.1 times. This demonstrated that even when considering all factors, clustering’s impact in the complete model remained statistically significant (Table 2).

## Discussion

This study aimed to identify the individual- and community-level factors of the plan to pre-lacteal feeding among mothers in Ethiopia. The proportion of plan to pre-lacteal feeding among mothers was 34% in the 2019 PMA-ET dataset. This proportion of pre-lacteal feeding reflects different individual and community factors that affect pre-lacteal feeding. Studies have found that interventions such as counseling and education programs for mothers and healthcare providers can lead to a decrease in the prevalence of pre-lacteal feeding [22]. However, the effectiveness of these interventions may vary depending on the context and cultural norms of each country.

At the individual level educational status and the wealth of the household were significantly associated with pre-lacteal feeding. At the community level, community wealth status was found to have a statistically significant association with the plan to pre-lacteal feeding.

In this study, mothers were not attended higher education were associated with plans to pre-lacteal feeding. The finding of this study is in line with previous studies and can also be justified by the fact that many studies have shown that maternal education level is associated with infant feeding practice [9, 23–25]. The possible reasons: Firstly, as mentioned earlier, mothers who did not attend higher education may not have had access to accurate and up-to-date information about infant feeding practices. This lack of awareness may have led them to believe that pre-lacteal feeding is necessary for their infant’s health. Secondly, cultural beliefs and practices may also play a role in this association. In some cultures, it is believed that newborns need other liquids or foods before breastfeeding to help them adjust to the outside world. Mothers who did not attend higher education may be more likely to follow these traditional practices or beliefs.

This study reveals that poor wealth index quintiles were less likely to plan to pre-lacteal feeding relative to the rich wealth category. A similar outcome is recognized in studies conducted in different countries [5, 26]. This could be related to the fact that mothers with poor wealth status may not access health services and counseling from healthcare providers on infant feeding practices. Contrary to this, studies evidenced that being in poor wealth status is preventive for pre-lacteal feeding. Because those of low socio-economic status could not afford the expensive pre-lacteal food like honey and ghee so the only available option to them is exclusive breastfeeding [9]. The possible reasons might be mothers from lower wealth quintiles may have limited access to alternative feeding options such as formula or other liquids, which may lead them to rely on exclusive breastfeeding. Secondly, mothers from lower wealth quintiles may have a stronger motivation to breastfeed exclusively due to financial constraints. Formula or other feeding options can be expensive, and exclusive breastfeeding may be a more cost-effective option for these mothers.

The strength of the current study includes the use of multilevel mixed effect analysis it accounts for the nested structure of data, where observations are nested within groups or clusters, allowing for the estimation of both within-group and between-group effects and the examination of individual and group-level predictors simultaneously. The study also has some limitations, it assumes that the effects of predictors are constant across all levels, which may not always be the case. It may be prone to bias if there is unobserved heterogeneity across groups or clusters.

## Conclusions

Individual and community-level factors were found to influence the decision to practice pre-lacteal feeding. Educational status and household wealth were significant individual-level factors associated with pre-lacteal feeding, whereas community wealth status was a significant community-level factor. Strengthening maternal education level and improving maternal income generating activities with the integration of maternal nutrition education on the benefits of exclusive breastfeeding and the risks associated with pre-lacteal feeding practice. Programs that aim to improve maternal education and financial stability may help to reduce the prevalence of pre-lacteal feeding. Additionally, community-level interventions that address poverty and inequality may also be effective in promoting appropriate breastfeeding practices. Further research is needed to identify the most effective strategies for addressing these factors and improving infant and young child feeding practices in Ethiopia.

## Data Availability

All the necessary data are included in the manuscript. The detailed information was found within the PMA report and gained the data set by requesting permission through the website https://datalab.pmadata.org/dataset.

## Abbreviations

AOR: Adjusted Odds Ratio
EAs: Enumeration Areas
IYCF: Infant and Young Child Feeding
SNNP: South Nation and Nationalities people
PMA-ET: Performance Monitoring for Action Ethiopia
